# Supplementary material for: Correlational Study of Aminopeptidase Activities between Left or Right Frontal Cortex versus the Hypothalamus, Pituitary, Adrenal Axis of Spontaneously Hypertensive Rats Treated with Hypotensive or Hypertensive Agents
Source: Int J Mol Sci. 2023 Nov 6;24(21):16007. doi: 10.3390/ijms242116007 (PMC10647763; doi:10.3390/ijms242116007)
Supplement: Supplementary file 1 [file ijms-24-16007-s001.zip › ijms-2625983-supplementary.pdf]

SUPPLEMENTARY MATERIAL: Table S1A, Table S1B, Table S2A, Table S2B, Table S3A and Table S3B

Table S1A: Positive or negative correlations between the angiotensinase activities analyzed in the right or left frontal cortices versus angiotensinase activities analyzed in hypothalamus, pituitary or adrenal gland in control SHR.

**FRONTAL CORTEX**		**HYPOTHALAMUS**						**PITUITARY**						**ADRENAL**					
		HAS	HCS	HGS	HAM	HCM	HGM	PAS	PCS	PGS	PAM	PCM	PGM	AAS	ACS	AGS	AAM	ACM	AGM
**RIGHT**	RAS	**−0.74**<br>***0.03***	−0.35	−0.49	−0.11	0.06	0.04	−0.13	−0.45	0.03	**−0.74**<br>***0.03***	−0.64	−0.03	−0.29	−0.43	−0.09	−0.14	−0.5	0.31
	RCS	−0.51	−0.23	−0.29	−0.34	−0.1	−0.26	−0.01	−0.25	0.24	−0.49	−0.56	0.23	−0.36	−0.49	−0.003	−0.58	**−0.80**<br>***0.01***	0.05
	RGS	−0.51	−0.15	−0.21	−0.21	0.03	−0.19	0.08	−0.20	0.38	−0.46	−0.40	0-33	−0.19	−0.32	−0.16	−0.5	−0.68	0.05
	RAM	−0.19	−0.42	−0.44	−0.15	−0.06	0.07	−0.61	−0.13	−0.08	−0.004	−0.12	0.004	−0.28	0.26	0.33	0.56	0.44	0.07
	RCM	0.27	−0.18	−0.13	−0.49	0.005	−0.21	−0.55	−0.08	0.1	0.17	−0.04	0.07	−0.42	−0.04	0.41	0.06	0.06	0.1
	RGM	0.34	−0.31	0.06	−0.1	−0.31	−0.31	−0.42	−0.06	0.11	0.26	0.25	0.51	−0.3	0.38	0.63	−0.22	0.04	−0.12
**LEFT**	LAS	−0.28	0.32	0.24	0.18	0.27	−0.28	0.34	0.03	0.07	−0.24	0.006	−0.03	0.43	−0.06	−0.29	0.02	0.02	−0.33
	LCS	−0.16	0.58	0.39	0.32	0.25	−0.12	0.65	0.44	0.11	0.13	0.26	−0.06	**0.75**<br>***0.03***	0.16	−0.58	0.22	0.27	−0.56
	LGS	0.42	0.07	0.24	**0.77**<br>***0.02***	0.03	0.67	0.18	−0.14	−0.64	0.03	0.41	−0.53	0.32	0.26	−0.04	0.34	0.52	0.17
	LAM	0.34	0.45	0.33	−0.28	−0.14	−0.4	0.22	0.32	−0.25	0.3	−0.04	−0.36	0.06	−0.42	0.1	−0.19	−0.12	0.18
	LCM	0.14	−0.36	−0.09	0.38	−0.05	0.57	−0.04	−0.38	−0.05	−0.19	0.13	0.12	−0.18	0.15	0.01	−0.25	−0.17	0.26
	LGM	0.55	0.58	0.63	−0.43	0.34	−0.53	0.36	0.47	**0.89**<br>***0.003***	0.56	0.50	0.62	0.24	0.06	−0.31	−0.55	−0.29	−0.61

Positive (black) or negative (red) correlations of soluble (S) or membrane-bound (M) angiotensinase activities between right (R) or left (L) frontal cortices versus angiotensinase activities of hypothalamus (H), pituitary (P) and adrenal (A) in CONTROL SHR. When significant, Pearson's correlation coefficients (r) and p values (italics) are indicated in bold. Soluble Alanyl-aminopeptidase (AS); membrane-bound Alanyl-aminopeptidase (AM); Soluble cystinyl-aminopeptidase (CS); membrane-bound cystinyl-aminopeptidase (CM); soluble glutamyl-aminopeptidase (GS); membrane-bound glutamyl-aminopeptidase (GM).

Table S1B: Positive or negative correlations between the angiotensinase activities analyzed in hypothalamus, pituitary or adrenal gland versus angiotensinase activities analyzed in hypothalamus, pituitary or adrenal gland in control SHR.

		HYPOTHALAMUS						PITUITARY						ADRENAL					
		HAS	HCS	HGS	HAM	HCM	HGM	PAS	PCS	PGS	PAM	PCM	PGM	AAS	ACS	AGS	AAM	ACM	AGM
HYPOTHALAMUS	HAS	1	0.58	**0.81** *0.01*	0.1	0.39	−0.02	0.34	0.17	0.19	0.48	**0.72** *0.04*	0.01	0.34	0.08	−0.17	−0.3	0.07	−0.25
	HCS		1	**0.89** *0.003*	0.08	0.57	−0.18	**0.84** *0.009*	0.54	0.42	0.46	0.62	−0.02	**0.78** *0.02*	−0.03	**−0.73** *0.03*	−0.19	0.03	−0.58
	HGS			1	0.19	0.59	−0.14	**0.72** *0.04*	0.38	0.42	0.46	**0.80** *0.01*	0.09	**0.72** *0.04*	0.1	−0.56	−0.29	0.05	−0.59
	HAM				1	0.25	**0.80** *0.01*	0.33	−0.1	−0.38	−0.06	0.52	−0.32	0.61	0.55	−0.39	0.56	0.69	−0.21
	HCM					1	0.16	0.35	−0.26	0.24	−0.26	0.40	−0.27	0.51	−0.18	−0.63	0.03	0.07	−0.28
	HGM						1	0.08	−0.3	−0.48	−0.24	0.19	−0.46	0.24	0.31	−0.29	0.54	0.5	0.18
PITUITARY	PAS							1	0.62	0.40	0.46	0.61	0.09	**0.84** *0.009*	0.17	**−0.86** *0.006*	−0.17	0.04	−0.6
	PCS								1	0.48	**0.89** *0.003*	0.44	0.47	0.49	0.42	−0.36	−0.09	0.14	−0.66
	PGS									1	0.43	0.36	**0.80** *0.01*	0.22	0.14	−0.4	−0.55	−0.38	−0.68
	PAM										1	0.62	0.51	0.39	0.54	−0.16	−0.12	0.23	−0.62
	PCM											1	0.25	**0.77** *0.02*	0.64	−0.51	0.05	0.47	**−0.75** *0.03*
	PGM												1	−0.04	0.44	0.03	−0.47	−0.26	−0.57
ADRENAL	AAS													1	0.44	**−0.82** *0.01*	0.2	0.5	**−0.74** *0.03*
	ACS														1	−0.11	0.43	**0.70** *0.05*	−0.64
	AGS															1	−0.03	−0.11	0.55
	AAM																1	**0.88** *0.003*	−0.04
	ACM																	1	−0.34
	AGM																		1

Positive (black) or negative (red) correlations of soluble (S) or membrane-bound (M) angiotensinase activities between hypothalamus (H), pituitary (P) and adrenal (A) versus angiotensinase activities of hypothalamus, pituitary and adrenal in CONTROL SHR. When significant, Pearson's correlation coefficients (r) and p values (italics) are indicated

in bold. Soluble Alanyl-aminopeptidase (AS); membrane-bound Alanyl-aminopeptidase (AM); Soluble cystinyl-aminopeptidase (CS); membrane-bound cystinyl-aminopeptidase (CM); soluble glutamyl-aminopeptidase (GS); membrane-bound glutamyl-aminopeptidase (GM).

Table S2A: Positive or negative correlations between the angiotensinase activities analyzed in the right or left frontal cortices versus angiotensinase activities analyzed in hypothalamus, pituitary or adrenal gland in captopril treated SHR.

**FRONTAL CORTEX**		**HYPOTHALAMUS**						**PITUITARY**						**ADRENAL**					
		HAS	HCS	HGS	HAM	HCM	HGM	PAS	PCS	PGS	PAM	PCM	PGM	AAS	ACS	AGS	AAM	ACM	AGM
**RIGHT**	RAS	0.13	0.08	−0.08	−0.47	−0.48	−0.57	0.21	−0.46	0.11	0.04	0.19	0.48	−0.1	−0.13	0.36	0.19	0.06	0.5
	RCS	−0.42	0.68	0.27	−0.12	0.15	0.002	0.27	−0.01	−0.15	−0.37	0.16	0.02	−0.18	−0.32	0.28	−0.02	−0.03	0.02
	RGS	−0.19	0.49	0.04	−0.42	−0.34	−0.41	0.33	−0.57	−0.16	−0.11	0.43	0.30	−0.4	−0.4	0.15	0.43	0.36	0.52
	RAM	−0.04	0.05	−0.56	0.26	0.18	0.44	0.08	0.04	−0.16	0.31	0.12	−0.27	0.54	0.34	0.12	0.34	0.38	0.26
	RCM	−0.1	−0.11	−0.06	−0.14	0.29	0.26	−0.21	0.23	−0.13	−0.34	−0.54	**−0.72** ***0.02***	0.43	0.19	−0.09	−0.15	−0.16	−0.36
	RGM	−0.07	0.13	**0.70** ***0.05***	0.37	0.55	0.39	−0.1	0.69	−0.04	−0.43	−0.33	−0.26	−0-13	−0.02	−0.1	−0.68	−0.6	**−0.82** ***0.01***
**LEFT**	LAS	−0.21	0.09	−0.56	0.67	0.38	0.58	−0.1	0.57	−0.23	0.42	−0.09	−0.15	0.58	0.56	0.38	−0.47	−0.32	−0.31
	LCS	−0.37	0.18	−0.37	−0.07	−0.34	−0.29	−0.5	−0.39	−0.27	0.54	0.08	−0.02	−0.11	−0.13	−0.08	−0.08	0.12	0.14
	LGS	−0.66	0.34	−0.41	0.22	0.06	0.14	−0.56	−0.09	−0.34	0.43	0.02	−0.38	−0.001	−0.13	−0.23	−0.11	0.17	−0.19
	LAM	0.1	0.24	−0.02	−0.08	−0.2	0.07	**0.70** ***0.05***	−0.23	−0.55	0.19	0.31	0.12	0.5	0.59	**0.72** ***0.02***	0.42	0.25	0.57
	LCM	0.48	−0.09	**0.89** ***0.003***	−0.19	−0.02	−0.06	0.4	0.11	−0.37	−0.5	−0.19	−0.07	−0.16	0.23	0.07	−0.16	−0.33	−0.3
	LGM	0.14	−0.17	0.12	0.11	0.22	0.37	0.09	0.35	−0.36	−0.06	−0.38	−0.48	0.64	0.65	0.26	−0.2	0.26−	0.27−

Positive (black) or negative (red) correlations of soluble (S) or membrane-bound (M) angiotensinase activities between right (R) or left (L) frontal cortices versus angiotensinase activities of hypothalamus (H), pituitary (P) and adrenal (A) in CAPTOPRIL SHR. When significant, Pearson's correlation coefficients (r) and p values (italics) are indicated in bold. Soluble Alanyl-aminopeptidase (AS); membrane-bound Alanyl-aminopeptidase (AM); Soluble cystinyl-aminopeptidase (CS); membrane-bound cystinyl-aminopeptidase (CM); soluble glutamyl-aminopeptidase (GS); membrane-bound glutamyl-aminopeptidase (GM).

Table S2B: Positive or negative correlations between the angiotensinase activities analyzed in hypothalamus, pituitary or adrenal gland versus angiotensinase activities analyzed in hypothalamus, pituitary or adrenal gland in captopril treated SHR.

		HYPOTHALAMUS						PITUITARY						ADRENAL					
		HAS	HCS	HGS	HAM	HCM	HGM	PAS	PCS	PGS	PAM	PCM	PGM	AAS	ACS	AGS	AAM	ACM	AGM
HYPOTHALAMUS	HAS	1	**−0.82** ***0.01***	0.34	−0.30	−0.52	−0.52	0.13	−0.08	0.2	0.07	−0.24	0.48	0.06	0.44	0.18	−0.18	−0.41	0.03
	HCS		1	0.02	0.24	0.45	0.48	0.37	0.02	−0.38	−0.23	0.47	−0.23	−0.15	−0.34	0.1	0.34	0.43	0.13
	HGS			1	−0.12	0.16	−0.004	0.3	0.31	−0.2	−0.68	−0.28	−0.08	−0.05	0.04	0.05	−0.38	−0.52	−0.52
	HAM				1	**0.71** ***0.04***	**0.85** ***0.007***	0.22	0.69	0.30	0.22	0.40	0.20	0.14	0.06	0.07	−0.03	0.08	−0.13
	HCM					1	**0.91** ***0.001***	0.13	**0.83** ***0.01***	0.25	−0.44	0.03	−0.3	0.05	−0.2	−0.09	−0.2	−0.1	−0.52
	HGM						1	0.27	**0.75** ***0.03***	0.08	−0.14	0.25	−0.20	0.24	0.03	0.08	−0.03	0.06	−0.28
PITUITARY	PAS							1	0.04	−0.09	−0.14	0.62	0.48	0.05	0.16	0.51	0.50	0.29	0.46
	PCS								1	0.33	−0.31	−0.26	−0.15	0.31	0.18	0.13	−0.58	−0.54	−0.69
	PGS									1	−0.06	0.09	0.46	−0.23	−0.37	−0.3	−0.02	−0.03	−0.07
	PAM										1	0.31	0.39	0.34	0.41	0.25	0.18	0.26	0.54
	PCM											1	0.61	−0.34	−0.27	0.05	**0.81** ***0.01***	**0.81** ***0.01***	**0.73** ***0.03***
	PGM												1	−0.19	0.03	0.25	0.35	0.21	0.57
ADRENAL	AAS													1	**0.84** ***0.009***	**0.73** ***0.03***	−0.36	−0.39	−0.03
	ACS														1	**0.76** ***0.02***	−0.34	−0.44	0.02
	AGS															1	−0.13	−0.27	0.27
	AAM																1	**0.94** ***0.0005***	**0.83** ***0.01***
	ACM																	1	**0.74** ***0.03***
	AGM																		1

Positive (black) or negative (red) correlations of soluble (S) or membrane-bound (M) angiotensinase activities between hypothalamus (H), pituitary (P) and adrenal (A) versus angiotensinase activities of hypothalamus, pituitary and adrenal in CAPTOPRIL SHR. When significant, Pearson's correlation coefficients (r) and p values (italics) are indicated in bold. Soluble Alanyl-aminopeptidase (AS); membrane-bound Alanyl-aminopeptidase (AM); Soluble cystinyl-aminopeptidase (CS); membrane-bound cystinyl-aminopeptidase (CM); soluble glutamyl-aminopeptidase (GS); membrane-bound glutamyl-aminopeptidase (GM).

Table S3A: Positive or negative correlations between the angiotensinase activities analyzed in the right or left frontal cortices versus angiotensinase activities analyzed in hypothalamus, pituitary or adrenal gland in L-NAME treated SHR.

**FRONTAL CORTEX**		**HYPOTHALAMUS**						**PITUITARY**						**ADRENAL**					
		HAS	HCS	HGS	HAM	HCM	HGM	PAS	PCS	PGS	PAM	PCM	PGM	AAS	ACS	AGS	AAM	ACM	AGM
**RIGHT**	RAS	0.67	−0.07	0.21	−0.16	−0.29	−0.20	−0.20	0.42	−0.14	−0.29	−0.60	−0.26	0.34	0.18	−0.06	−0.07	−0.25	−0.57
	RCS	0.28	−0.02	0.16	0.08	−0.15	0.12	−0.27	0.04	−0.57	−0.56	−0.60	−0.58	−0.11	−0.26	−0.11	−0.22	−0.39	−0.05
	RGS	0.4	−0.05	0.15	−0.06	−0.31	−0.02	−0.27	0.17	−0.52	−0.42	−0.64	−0.46	−0.09	−0.27	−0.16	−0.25	−0.46	−0.01
	RAM	−0.46	−0.07	−0.15	0.27	0.21	0.30	0.16	−0.06	−0.24	**−0.83**<br>***0.01***	−0.13	−0.69	0.23	0.13	−0.26	−0.38	−0.3	−0.18
	RCM	0.02	−0.59	−0.49	−0.52	−0.52	−0.49	0.54	**0.73**<br>***0.03***	0.26	−0.2	−0.24	−0.43	**0.70**<br>***0.05***	0.65	−0.3	−0.63	−0.67	−0.41
	RGM	0.36	0.02	0.25	0.11	−0.07	0.11	−0.16	0.2	−0.35	−0.60	−0.42	−0.44	0.16	−0.18	−0.3	−0.09	−0.24	−0.22
**LEFT**	LAS	0.22	**0.74**<br>***0.03***	**0.78**<br>***0.02***	0.47	0.43	0.55	**−0.78**<br>***0.02***	−0.44	−0.51	−0.68	−0.68	−0.05	−0.37	−0.34	0.19	0.32	0.14	−0.19
	LCS	−0.59	−0.26	−0.41	−0.25	−0.2	−0.14	0.2	0.05	−0.15	−0.24	−0.11	−0.1	0.05	0.09	−0.41	**−0.71**<br>***0.04***	−0.65	0.25
	LGS	−0.35	−0.32	−0.40	−0.28	−0.33	−0.25	−0.09	−0.08	−0.45	−0.004	−0.25	−0.03	−0.17	−0.05	−0.29	**−0.74**<br>***0.03***	−0.65	0.23
	LAM	−0.23	0.56	0.42	0.53	0.57	0.49	−0.66	**−0.77**<br>***0.02***	−0.43	−0.21	−0.09	0.25	−0.47	−0.31	0.2	0.25	0.38	−0.02
	LCM	0.12	−0.23	−0.28	−0.43	−0.24	−0.42	0.26	0.33	0.56	0.43	−0.1	0.1	0.35	**0.83**<br>***0.01***	0.51	−0.05	−0.02	−0.51
	LGM	0.01	−0.04	−0.06	0.29	0.1	0.31	−0.1	−0.33	−0.36	−0.16	−0.23	**−0.80**<br>***0.01***	−0.42	−0.06	0.68	0.09	0.09	0.01

Positive (black) or negative (red) correlations of soluble (S) or membrane-bound (M) angiotensinase activities between right (R) or left (L) frontal cortices versus angiotensinase activities of hypothalamus (H), pituitary (P) and adrenal (A) in L-NAME SHR. When significant, Pearson's correlation coefficients (r) and p values (italics) are indicated in bold. Soluble Alanyl-aminopeptidase (AS); membrane-bound Alanyl-aminopeptidase (AM); Soluble cystinyl-aminopeptidase (CS); membrane-bound cystinyl-aminopeptidase (CM); soluble glutamyl-aminopeptidase (GS); membrane-bound glutamyl-aminopeptidase (GM).

Table S3B: Positive or negative correlations between the angiotensinase activities analyzed in hypothalamus, pituitary or adrenal gland versus angiotensinase activities analyzed in hypothalamus, pituitary or adrenal gland in L-NAME treated SHR.

		HYPOTHALAMUS						PITUITARY						ADRENAL					
		HAS	HCS	HGS	HAM	HCM	HGM	PAS	PCS	PGS	PAM	PCM	PGM	AAS	ACS	AGS	AAM	ACM	AGM
HYPOTHALAMUS	HAS	1	0.05	0.4	−0.28	−0.39	−0.38	−0.45	0.36	−0.09	0.29	−0.46	0.37	0.11	−0.07	−0.02	0.19	−0.01	−0.39
	HCS		1	**0.91**<br>***0.001***	0.66	**0.76**<br>***0.02***	**0.76**<br>***0.02***	−0.58	−0.56	−0.09	−0.47	−0.15	0.27	−0.42	−0.45	0.26	**0.79**<br>***0.01***	0.63	0.06
	HGS			1	0.54	0.56	0.57	**−0.70**<br>***0.05***	−0.37	−0.18	−0.4	−0.28	0.37	−0.3	−0.49	0.11	**0.77**<br>***0.02***	0.56	−0.09
	HAM				1	**0.92**<br>***0.001***	**0.94**<br>***0.0005***	−0.41	**−0.83**<br>***0.01***	−0.37	−0.58	0.24	−0.24	−0.43	−0.55	0.21	0.63	**0.75**<br>***0.03***	0.07
	HCM					1	**0.92**<br>***0.001***	−0.27	**−0.74**<br>***0.03***	−0.07	−0.56	0.31	−0.06	−0.29	−0.33	0.24	**0.70**<br>***0.05***	**0.80**<br>***0.01***	0.02
	HGM						1	−0.34	**−0.81**<br>***0.01***	−0.28	−0.65	0.14	−0.27	−0.52	−0.52	0.31	0.64	0.66	0.23
PITUITARY	PAS							1	0.55	**0.71**<br>***0.04***	0.20	0.56	−0.19	0.53	0.52	−0.21	−0.33	−0.25	0.16
	PCS								1	0.57	0.31	−0.07	0.2	**0.77**<br>***0.02***	0.55	−0.45	−0.43	−0.57	−0.27
	PGS									1	0.29	0.43	0.34	0.59	0.6	−0.04	0.16	0.11	−0.1
	PAM										1	0.24	0.44	−0.001	0.15	0.13	−0.1	−0.06	0.15
	PCM											1	0.11	0.17	−0.06	−0.22	0.23	0.48	0.29
	PGM												1	0.15	−0.06	−0.28	0.3	0.21	−0.04
ADRENAL	AAS													1	**0.71**<br>***0.04***	−0.54	−0.3	−0.24	−0.62
	ACS														1	0.09	−0.39	−0.33	−0.61
	AGS															1	0.35	0.33	0.01
	AAM																1	**0.90**<br>***0.002***	0.06
	ACM																	1	−0.01
	AGM																		1

Positive (black) or negative (red) correlations of soluble (S) or membrane-bound (M) angiotensinase activities between hypothalamus (H), pituitary (P) and adrenal (A) versus angiotensinase activities of hypothalamus, pituitary and adrenal in L-NAME SHR. When significant, Pearson's correlation coefficients (r) and p values (italics) are indicated in bold. Soluble Alanyl-aminopeptidase (AS); membrane-bound Alanyl-aminopeptidase (AM); Soluble cystinyl-aminopeptidase (CS); membrane-bound cystinyl-aminopeptidase (CM); soluble glutamyl-aminopeptidase (GS); membrane-bound glutamyl-aminopeptidase (GM).
